# Select HDAC Inhibitors Enhance Osteolysis and Bone Metastasis Outgrowth but Can Be Mitigated With Bisphosphonate Therapy

**DOI:** 10.1002/jbm4.10694

**Published:** 2023-01-25

**Authors:** Miranda E Clements, Lauren Holtslander, Joshua R Johnson, Rachelle W Johnson

**Affiliations:** ^1^ Program in Cancer Biology Vanderbilt University Nashville TN USA; ^2^ Vanderbilt Center for Bone Biology, Department of Medicine, Division of Clinical Pharmacology Vanderbilt University Medical Center Nashville TN USA; ^3^ Department of Medicine, Division of Clinical Pharmacology Vanderbilt University Medical Center Nashville TN USA

**Keywords:** BREAST CANCER, HISTONE DEACETYLASE INHIBITORS, BONE METASTASIS

## Abstract

Breast cancer has a high predilection for spreading to bone with approximately 70% of patients who succumb to disease harboring bone disseminated tumor cells. Despite this high prevalence, treatments for bone metastatic breast cancer predominantly manage morbidities, including pain and hypercalcemia, rather than reducing bone metastasis incidence or growth. Histone deacetylase inhibitors (HDACi), including panobinostat, entinostat, and valproic acid, typically slow primary tumor progression and are currently in clinical trials for the treatment of many cancers, including primary and metastatic breast cancer, but their effects on bone metastatic disease have not been examined in preclinical models. We report that treatment with the HDACi panobinostat, but not entinostat or valproic acid, significantly reduced trabecular bone volume in tumor‐naïve mice, consistent with previous reports of HDACi‐induced bone loss. Surprisingly, treatment with entinostat or panobinostat, but not valproic acid, increased tumor burden and incidence in an experimental model of breast cancer bone metastasis. In vitro, multiple HDACi stimulated expression of pro‐osteolytic genes in breast tumor cells, suggesting this may be a mechanism by which HDACi fuel tumor growth. In support of this, combination therapy of panobinostat or entinostat with the antiresorptive bisphosphonate zoledronic acid prevented bone metastatic progression; however, the addition of zoledronic acid to panobinostat therapy failed to fully correct panobinostat‐induced bone loss. Together these data demonstrate that select HDACi fuel bone metastatic growth and provide potential mechanistic and therapeutic avenues to offset these effects. © 2022 The Authors. *JBMR Plus* published by Wiley Periodicals LLC on behalf of American Society for Bone and Mineral Research.

## Graphical abstract

Select histone deacetylase inhibitors (HDACi) reduce primary breast tumor growth but increase breast tumor burden in bone. The pro‐tumorigenic effect of HDACi in the bone metastatic site is reversible if combined with bisphosphonate therapy.

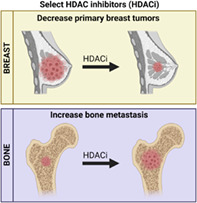

## Introduction

Breast cancer is the second‐leading cause of cancer deaths in women predominantly because of the metastatic spread of breast cancer cells to distant sites. Bone is the most common site of metastasis in patients with breast cancer^(^ ^1^ ^)^ and occurs in approximately 60% of metastatic estrogen receptor‐positive (ER^+^) patients and 40% of metastatic triple negative breast cancer (TNBC) patients.^(^ ^2^, ^3^ ^)^ Despite the high prevalence of bone metastasis, relatively few therapeutic options to effectively target bone metastases exist, and thus metastatic breast cancer remains incurable.

Given the central role of epigenetic modifications in driving tumor progression and metastasis, there has been significant interest in the development and use of epigenetic therapies for cancer management. Histone deacetylation is a major regulator of abnormal gene expression in tumor cells, and thus histone deacetylase inhibitors (HDACi) have been heavily pursued for their ability to reprogram tumor cells. Several HDACi have been approved by the FDA for hematological malignancies,^(^ ^4^ ^)^ and many HDACi are currently being tested in different combination therapies for their use in solid tumors, including metastatic breast cancer.

Of critical importance to bone metastatic disease, many cancer therapies have been shown to negatively impact bone health, resulting in increased risk of skeletal‐related events, morbidities, and mortality.^(^ ^5^ ^)^ The HDACi valproic acid is FDA‐approved for long‐term treatment of epilepsy, bipolar disorder, and migraines,^(^ ^6^ ^)^ but has been shown in several clinical studies to reduce bone mineral density, bone mass, and bone formation in patients.^(^ ^7^, ^8^, ^9^ ^)^ A small clinical study indicates that valproic acid treatment also increases fracture risk in both children and adults.^(^ ^10^, ^11^, ^12^ ^)^ Several preclinical studies suggest that the newer generation HDACi vorinostat negatively impacts bone homeostasis in non‐tumor‐inoculated mice through increased osteoclast activity and decreased osteoblast number, resulting in reduced bone volume.^(^ ^13^, ^14^ ^)^ Because elevated bone resorption results in the release of tumor‐promoting growth factors such as TGF‐β from the mineralized bone matrix,^(^ ^15^ ^)^ an outstanding question in the field is what impact HDACi‐mediated bone destruction will have on the progression of bone‐disseminated tumor cells. Thus, there is a critical need to investigate the effects of HDACi on normal and particularly tumor‐bearing bones.

## Materials and Methods

### Cells

Human MCF7 breast cancer cells were obtained from ATCC (Manassas, VA, USA). Human bone‐metastatic MDA‐MB‐231 cells (MDA‐MD‐231b)^(^ ^16^, ^17^ ^)^ were established from the bone clone generated by the Rhoades (Sterling) laboratory. Murine 4T1BM2 bone metastatic cells^(^ ^18^ ^)^ were gifted by Dr Normand Pouliot at the Peter MacCallum Cancer Centre. All cells were cultured in DMEM supplemented with 10% fetal bovine serum (FBS) and penicillin/streptomycin (P/S). All human cell lines were recently reauthenticated by ATCC. Cell lines are regularly tested for mycoplasma contamination.

### Animals

All experiments were performed following the relevant guidelines and regulations of the Animal Welfare Act and the Guide for the Care and Use of Laboratory Animals and were approved by the Institutional Animal Care and Use Committee (IACUC) at Vanderbilt University. Mice were group‐housed under specific pathogen‐free conditions, housed in a facility with a constant temperature of 23°C and 12‐hour light/dark cycle, and had *ad libitum* access to chow and water.

For intracardiac inoculation studies, female mice were inoculated with 1 × 10^5^ tumor cells as previously described^(^ ^19^, ^20^ ^)^ (*n* = 10 mice injected per group). Specifically, 4‐ to 6‐week‐old female athymic nude mice (Jackson Laboratory, Bar Harbor, ME, USA; catalog no. 7850) were used for the human MDA‐MB‐231b models. For the 4T1BM2 models, 4‐ to 6‐week‐old female BALB/c mice (Envigo Corp, Indianapolis, IN, USA; catalog no. 4702) were used. For studies using zoledronic acid, mice were given zoledronic acid (0.2 mg/kg; Selleckchem, Radnor, PA, USA; catalog no. S1314) via tail vein injection 24 hours before tumor cell inoculation. Treatment with vehicle (7.5% DMSO+10% HPBCD in sterile water), entinostat (10 mg/kg; Selleckchem, catalog no. S1053), panobinostat (5 mg/kg; Selleckchem, catalog no. S1030), or valproic acid (2 mg/kg, Sigma, St. Louis, MO, USA; catalog no. PHR1061) was initiated 24 hours post tumor cell inoculation and given 5 days per week by i.p. injection until euthanization. For each drug, dosages were chosen based on the IC50 and those previously reported in the literature to alter in vivo tumor growth. Mice were randomly allocated to treatment groups. Mice were euthanized at 4 weeks (MDA‐MB‐231b +/− HDAC inhibitor and zoledronic acid [ZA]), 3 weeks (4T1BM2 +/− valproic acid [VPA]), and 4 weeks (MDA‐MB‐231b +/− VPA). For each cohort, a total of *n* = 10 mice were originally injected. The number of mice indicated in the figure legends (*n* = 6–10 mice per group) represents the number of mice included in the final analysis. Mice that (i) died during intracardiac inoculation, (ii) became moribund and had to be euthanized early but had no evidence of tumor burden, or (iii) were found deceased were not included in the analysis.

### RNA extraction and real‐time qPCR

Cells were harvested for real‐time qPCR as previously described.^(^ ^20^ ^)^ Briefly, cells grown in monolayer were harvested in TRIzol (Thermo Fisher Scientific, Waltham, MA, USA), extracted, purified, and DNase treated (TURBO DNA‐free kit, Thermo Fisher Scientific), and cDNA synthesized (1000 ng RNA, iScript cDNA Synthesis Kit, Bio‐Rad, Hercules, CA, USA) per the manufacturer's instructions. Real‐time PCR was performed using iTaq Universal SYBR Green Supermix (Bio‐Rad) on a QuantStudio 5 (Thermo Fisher Scientific) with the following conditions: 2 minutes at 50°C, 10 minutes at 95°C, (15 seconds at 95°C, 1 minute 60°C) ×40 cycles followed by dissociation curve (15 seconds 95°C, 1 minute 60°C, 15 second 95°C). Human primers for *B2M*,^(^ ^20^, ^21^ ^)^ *HPRT1*,^(^ ^20^, ^21^ ^)^ and *PTHLH* ^(^ ^22^ ^)^ were previously published. Human primers for *GLI2* were designed using PrimerBlast (NCBI) against the human genome and validated by dissociation: *GLI2* (F: ATCAATCCTGTGGCCTACCA, R: TGACTCGCTGTTCTGCTTGT).

### HDAC inhibitor treatment

Cells were seeded in a 6‐well plate at 2 × 10^5^ cells/well for RNA analysis. The following day, cells were treated with vehicle (DMSO) or entinostat (5 mM; Selleckchem, catalog no. S1053) for 24 hours in full serum medium. Cells were harvested for RNA in TRIzol (Thermo Fisher Scientific) as discussed above.

### In vivo imaging

After injection of MDA‐MB‐231b cells expressing luciferase, metastatic tumor growth was monitored using the IVIS Spectrum instrument. Mice were injected with 150 mg/kg luciferin, and luminescence imaging was initiated 10 minutes after luciferin injection.

### Micro‐computed tomography (microCT)

Ex vivo micro‐CT was performed on the proximal tibia using the Scanco μCT 50 (Scanco Medical, Bruttisellen, Switzerland). Scans were initiated from the proximal end of the metaphyseal growth plate and progressed 200 slices distal. Tibias were scanned at 7 μm voxel resolution, 55‐kV voltage, and 200 μA current. Scans were reconstructed and analyzed using the Scanco Medical Imaging Software to determine the bone volume/total volume (BV/TV), trabecular number, thickness, and separation. The most distal slice of the proximal tibial growth plate was used as a reference slice, and analysis was set to begin 20 slices distal from this point. A 100‐slice region of interest was selected for analysis.

### Histology

Hindlimbs were dissected and fixed in 10% formalin for 48 hours and decalcified in EDTA (20% pH 7.4) solution for 72 hours. Decalcified bones were embedded in paraffin and 5‐μm thick sections were prepared for staining. Hematoxylin and eosin (H&E) staining was performed as previously described.^(^ ^20^ ^)^ H&E‐stained sections were analyzed by histomorphometry in the proximal secondary spongiosa using the OsteoMeasure software (Osteometrics, Decatur, GA, USA). Histological analysis of H&E‐stained tibiae was performed blinded by the authors and in some studies by an ACVP board‐certified veterinary anatomic pathologist who has specific expertise in mouse models of breast cancer. The entire length of the tibia was analyzed for the presence of tumor. Specifically, tumor cells in the bone were identified based on abnormal features such as prominent nucleoli, increased mitotic rate, large nuclei, high nuclear/cytoplasmic area, epithelial morphology, spindly cells that do not resemble normal bone cells (eg, osteoblasts), and cells that disrupt the normal architecture of the bone (growth plate, cortical bone).

### Tumor incidence

The lower portion of the mice including the hindlimbs were analyzed for bioluminescence signal using the Living Image Software (IVIS Imaging System, PerkinElmer, Waltham, MA, USA). The background bioluminescence signal from this region of interest (ROI) was determined using non‐tumor‐bearing mice injected with luciferase. The average radiant efficiency from this ROI was determined to be 3.2 × 10^8^ [p/s]/[μW/cm^2^]. Using this background bioluminescence value, mice were scored for the absence (sum bioluminescence less than 3.2 × 10^8^ [p/s]/[μW/cm^2^]) or presence (sum bioluminescence above 3.2 × 10^8^ [p/s]/[μW/cm^2^]) of bone metastasis. From this analysis, 1/10 vehicle‐treated (10%), 6/11 entinostat‐treated (54%), and 4/8 panobinostat‐treated (50%) mice were determined to harbor overt bone metastases, but this does not preclude the presence of disseminated tumor cells in the bone marrow. These results were confirmed by manual inspection of IVIS images, which confirmed higher signal specifically within the bone indicating the presence of bone metastases. The graphs presented in Fig. 2*B* are presented as the % of mice with the absence or presence of overt bone metastases.

### Statistical methods

For all studies, *n* per group is as indicated in the figure legend and the bar graphs indicate the mean of the data and error bars indicate standard error of the mean. The Prism software (GraphPad, La Jolla, CA, USA) was used to generate all graphs and statistical analyses. All in vitro and in vivo assays were analyzed for statistical significance using a Mann–Whitney *U* test where *p* < 0.05 was considered statistically significant and *p < 0.05, **p < 0.01, ***p < 0.001, ****p < 0.0001.

## Results

### Panobinostat, but not entinostat, results in bone loss and altered bone microarchitecture

**Fig. 1:**
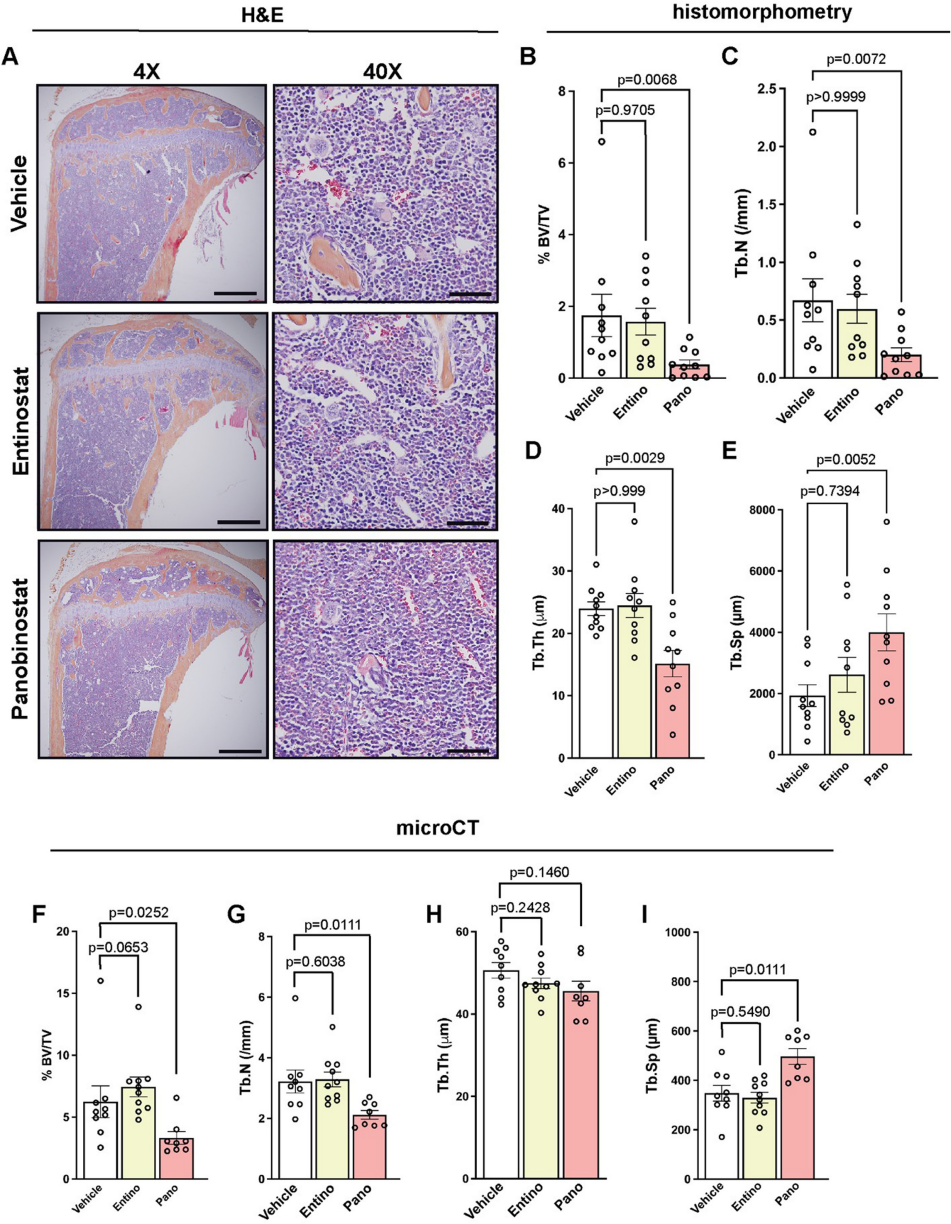
Panobinostat, but not entinostat, results in bone loss and altered bone microarchitecture. (*A*) Representative hematoxylin and eosin (H&E) images of tibias from naïve mice treated with vehicle, entinostat, or panobinostat (*n* = 10 mice/group). 4×, scale bar = 500 μM; 40×, scale bar = 100 μM. (*B*–*E*) Histomorphometric analysis of (*B*) bone volume/total volume (%BV/TV), (*C*) trabecular number (Tb.N), (*D*) trabecular thickness (Tb.Th), and (*E*) trabecular spacing (Tb.Sp) of mice described in (*A*). (*F*–*I*) Micro‐CT analysis of (*F*) bone volume/total volume (%BV/TV), (*G*) trabecular number, (*H*) trabecular thickness, and (*I*) trabecular spacing of mice described in (*A*). All bar graphs indicate the group mean and error bars indicate standard error of the mean.

We first sought to investigate the effects of the HDACi entinostat or panobinostat on trabecular bone structure, since both drugs are currently or have recently been investigated in breast cancer clinical trials. After 4 weeks of treatment with HDACi in tumor‐naïve mice, metaphyseal trabecular bone microarchitecture was assessed by histomorphometry in the proximal tibia and by micro‐CT in the distal femur. Panobinostat treatment resulted in a significant reduction in bone volume (1.88‐ to 4.6‐fold) and trabecular number (1.52‐ to 1.66‐fold, *p* = 0.0068–0.0072) and a significant increase in trabecular separation (1.39‐ to 1.42‐fold, *p* = 0.0052–0.0111) in both the tibia and femur, suggesting that panobinostat results in trabecular bone loss and altered bone microarchitecture (Fig. 1*A*–*I*). Notably, mice treated with entinostat showed no change in tibial or femoral trabecular bone volume or microarchitecture (Fig. 1*A*–*I*). A trend toward an increase in trabecular bone volume (1.19‐fold, *p* = 0.0653) was observed in the distal femur by micro‐CT; however, given the lack of change in any other trabecular parameters, this effect is likely attributable to statistical chance rather than biological differences.

### Entinostat and panobinostat treatment increases tumor burden in bone

**Fig. 2:**
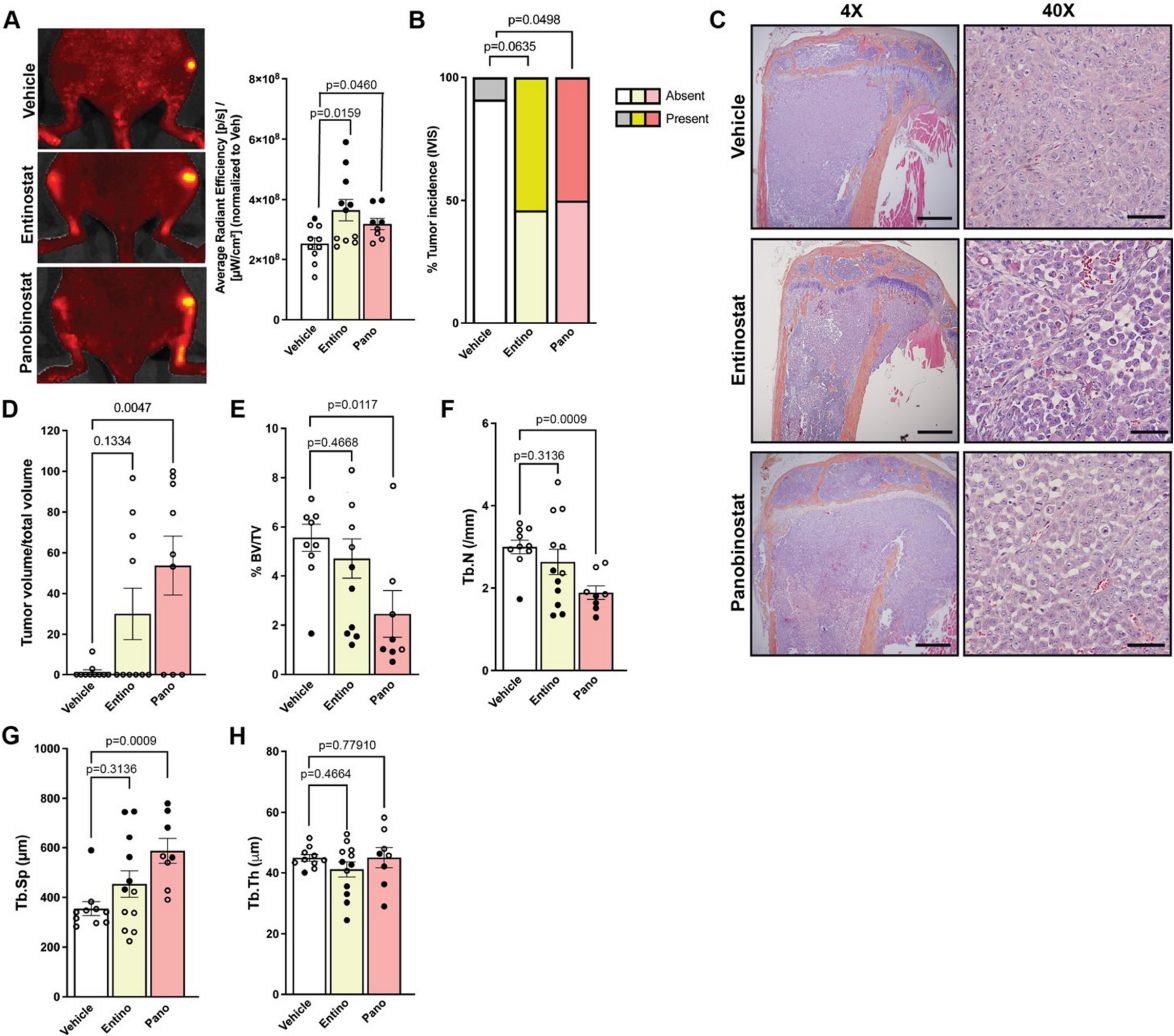
Entinostat and panobinostat treatment increase tumor burden in bone. (*A*) Representative in vivo bioluminescence images and quantitation at endpoint of luciferase‐expressing MDA‐MB‐231b‐inoculated mice treated with vehicle, entinostat, or panobinostat (*n* = 10 mice/group). (*B*) Analysis of tumor incidence of tibias and femurs from images described in (*A*). (*C*) Representative hematoxylin and eosin (H&E) images of tibias from mice described in (*A*). 4×, scale bar = 500 μM; 40×, scale bar = 100 μM. (*D*) Histological analysis of tumor cells from the bone marrow of mice described in (*A*). (*E*–*H*) Micro‐CT analysis of (*E*) bone volume/total volume (%BV/TV), (*F*) trabecular number, (*G*) trabecular spacing, and (*H*) trabecular thickness of tibiae from mice described in (*A*). All bar graphs indicate the group mean and error bars indicate standard error of the mean. For all graphs, mice harboring overt bone metastases are indicated by solid black points. Mice presented in Fig. 1 represent tumor‐naïve mice that can be directly compared with the mice presented herein as they were all included in the same experiment.

Because we previously demonstrated that entinostat reduces primary breast tumor growth^(^ ^23^ ^)^ and entinostat therapy did not cause bone loss in tumor‐naïve mice, we hypothesized that entinostat may effectively reduce tumor burden in bone. Panobinostat reduces primary breast tumor growth similar to entinostat;^(^ ^23^ ^)^ however, because panobinostat significantly reduced trabecular number, we hypothesized that the changes in the bone niche may accelerate tumor progression in bone. To test these hypotheses, mice were inoculated with MDA‐MB‐231b (bone metastatic clone; ER−^(^ ^16^, ^17^ ^)^) by intracardiac injection, and the next day, HDACi therapy (entinostat or panobinostat) was initiated. To assess tumor progression in bone, we performed in vivo bioluminescence imaging for luciferase‐positive MDA‐MB‐231b tumor cells. Surprisingly, both entinostat and panobinostat treatment resulted in significantly higher tumor burden compared with vehicle‐treated mice (1.44‐fold, *p* = 0.0159 and 1.26‐fold, *p* = 0.0460, respectively; mice harboring overt bone metastases are indicated by solid black points) (Fig. 2*A*). Tumor incidence was also significantly increased in panobinostat and to a lesser extent entinostat‐treated mice compared with vehicle‐treated mice (up to 18‐fold, *p* = 0.0498–0.0635) (Fig. 2*B*). Histologic analysis of tumor burden confirmed a significant increase in tumor burden in panobinostat‐treated mice and a non‐significant trend toward an increase in tumor burden in entinostat‐treated mice (Fig. 2*C*, *D*). To assess whether the increase in bone tumor burden observed in HDACi‐treated mice resulted in tumor‐induced osteolysis and bone loss, we analyzed trabecular bone volume by micro‐CT. Although we observed significantly higher tumor burden in the bones of entinostat‐treated mice compared with vehicle‐treated control mice by IVIS, there was no change in trabecular bone volume after treatment with entinostat (Fig. 2*E*). In contrast, panobinostat‐treated mice had significantly reduced trabecular bone volume (2.26‐fold, *p* = 0.0117) and trabecular number (1.59‐fold, *p* = 0.0009), and significantly increased trabecular spacing (1.65‐fold, *p* = 0.0009), with no change in trabecular thickness (Fig. 2*F*–*H*). Notably, within each treatment group, mice harboring overt bone metastases (indicated by black points in the graphs) as determined by IVIS exhibited relatively lower bone volume, trabecular number, and trabecular thickness along with higher trabecular spacing compared with mice not harboring overt bone metastases. Overall, entinostat treatment enhances tumor growth in the bone without altering bone microarchitecture. Conversely, panobinostat treatment results in significant bone loss in the presence of tumor cells and fuels tumor growth in the bone metastatic site. These findings are in stark contrast to the tumor‐suppressive effect of both entinostat and panobinostat in the primary tumor site, where both HDACi significantly slow primary breast tumor growth.^(^ ^23^ ^)^

### Valproic acid does alter bone homeostasis or tumor growth in the context of bone metastasis

**Fig. 3:**
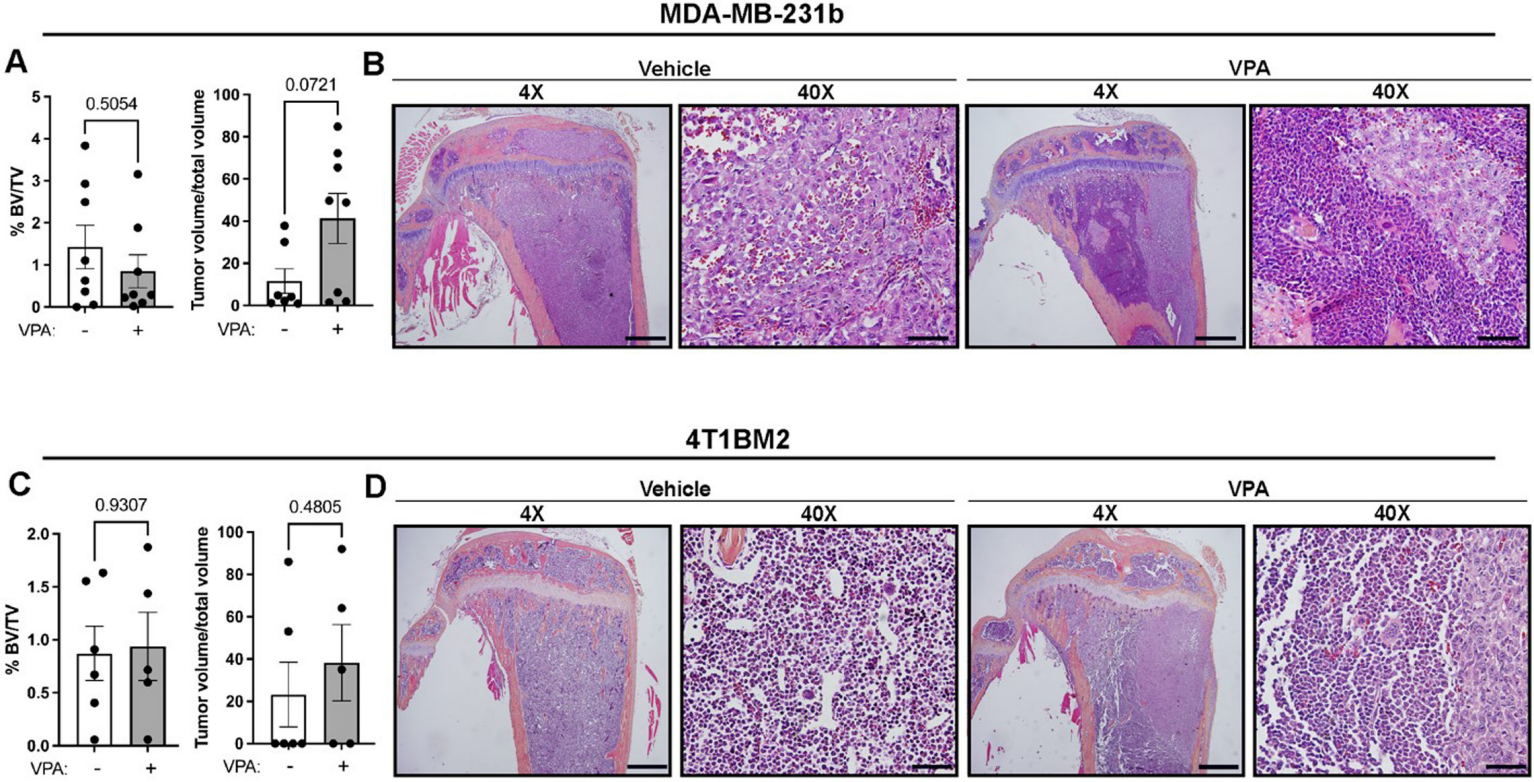
Valproic acid (VPA) does alter bone homeostasis or tumor growth in the context of bone metastasis. (*A*) Histomorphometric analysis of bone volume/total volume (%BV/TV) and tumor volume/total volume of tibias from MDA‐MB‐231b‐incoulated mice treated with VPA (*n* = 8 mice/group). (*B*) Representative hematoxylin and eosin (H&E) images of tibias from mice described in (*A*). 4×, scale bar = 500 μM; 40×, scale bar = 100 μM. (*C*) Histomorphometric analysis of bone volume/total volume (%BV/TV) and tumor volume/total volume of tibias from 4T1BM2‐incoulated mice treated with VPA (*n* = 5–6 mice/group). (*D*) Representative hematoxylin and eosin (H&E) images of tibias from mice described in (*C*). 4×, scale bar = 500 μM; 40×, scale bar = 100 μM. All bar graphs indicate the group mean and error bars indicate standard error of the mean.

Because we observed significant bone loss and enhanced tumor burden after panobinostat treatment in the bone metastatic setting, we investigated whether this phenomenon is observed with other HDACi known to disrupt bone homeostasis. The HDACi valproic acid (VPA) was previously shown to reduce physiological bone volume in naïve SCID/NCr mice (BALB/c background) and C57BL/6 mice.^(^ ^13^, ^14^ ^)^ We therefore investigated whether valproic acid alters bone volume in athymic nude mice and BALB/c mice or tumor progression in bone. Valproic acid treatment did not significantly alter trabecular bone volume or tumor burden as assessed by histomorphometry in mice inoculated with human MDA‐MB‐231b breast cancer cells (Fig. 3*A*, *B*) or a bone‐metastatic variant of 4T1 mouse mammary carcinoma cells (4T1BM2;^(^ ^18^ ^)^ Fig. 3*C*, *D*). Collectively, these data suggest that although some HDAC inhibitors result in bone loss, these effects are inhibitor‐specific and do not necessarily correspond to changes in metastatic progression.

### HDACi induce pro‐osteolytic genes in breast tumor cells

**Fig. 4:**
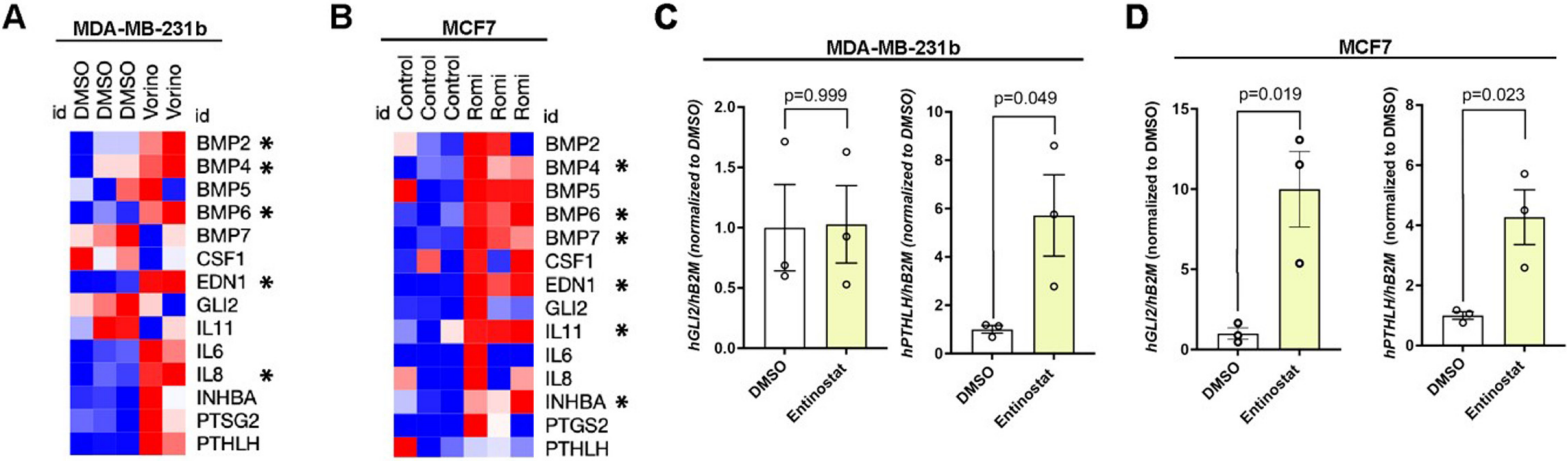
Entinostat induces pro‐osteolytic genes in breast tumor cells. (*A*, *B*) Heatmap showing mRNA levels of osteolytic‐associated factors in (*A*) MDA‐MB‐231 cells treated with vorinostat for 30 hours from GSE60125 and (*B*) MCF7 cells treated with romidepsin for 24 hours from GSE74036. (*C*, *D*) mRNA levels of human (h) *GLI2* and *PTHLH* in (*C*) MDA‐MB‐231b and (*D*) MCF7 cells treated with 5 μM entinostat or DMSO (vehicle control) for 24 hours, normalized to human beta 2 microglobulin (β2M) housekeeping control gene. All bar graphs indicate the group mean and error bars indicate standard error of the mean.

Using two publicly available data sets, we found that MDA‐MB‐231 cells treated with the HDACi vorinostat for 30 hours (GSE60125) and human MCF7 breast cancer cells treated with the HDACi romidepsin for 24 hours (GSE74036) showed significantly higher levels of multiple factors known to promote osteolysis (Fig. 4*A*, *B*). The pro‐osteolytic factors *BMP4* (up to 1.88‐fold, *p* = 0.0092–0.045), *BMP6* (up to 2.94‐fold, *p* = 0.0011–0.027), and *EDN1* (up to 3.09‐fold, *p* = 0.0005–0.0022) were significantly upregulated in both data sets. We also investigated whether the expression of well‐known osteolytic factors *GLI2* and *PTHLH* ^(^ ^15^, ^24^, ^25^, ^26^ ^)^ were elevated after entinostat treatment. *PTHLH* but not *GLI2* was significantly increased in MDA‐MB‐231b cells, and *GLI2* and *PTHLH* were both significantly increased in MCF7 cells after 24 hours of entinostat treatment (up to 9.7‐fold, *p* = 0.019–0.049) (Fig. 4*C*, *D*). Together, these findings suggest that multiple HDACi induce pro‐osteolytic factors in tumor cells.

### Combination treatment of entinostat or panobinostat with bisphosphonate prevents bone metastatic progression

**Fig. 5:**
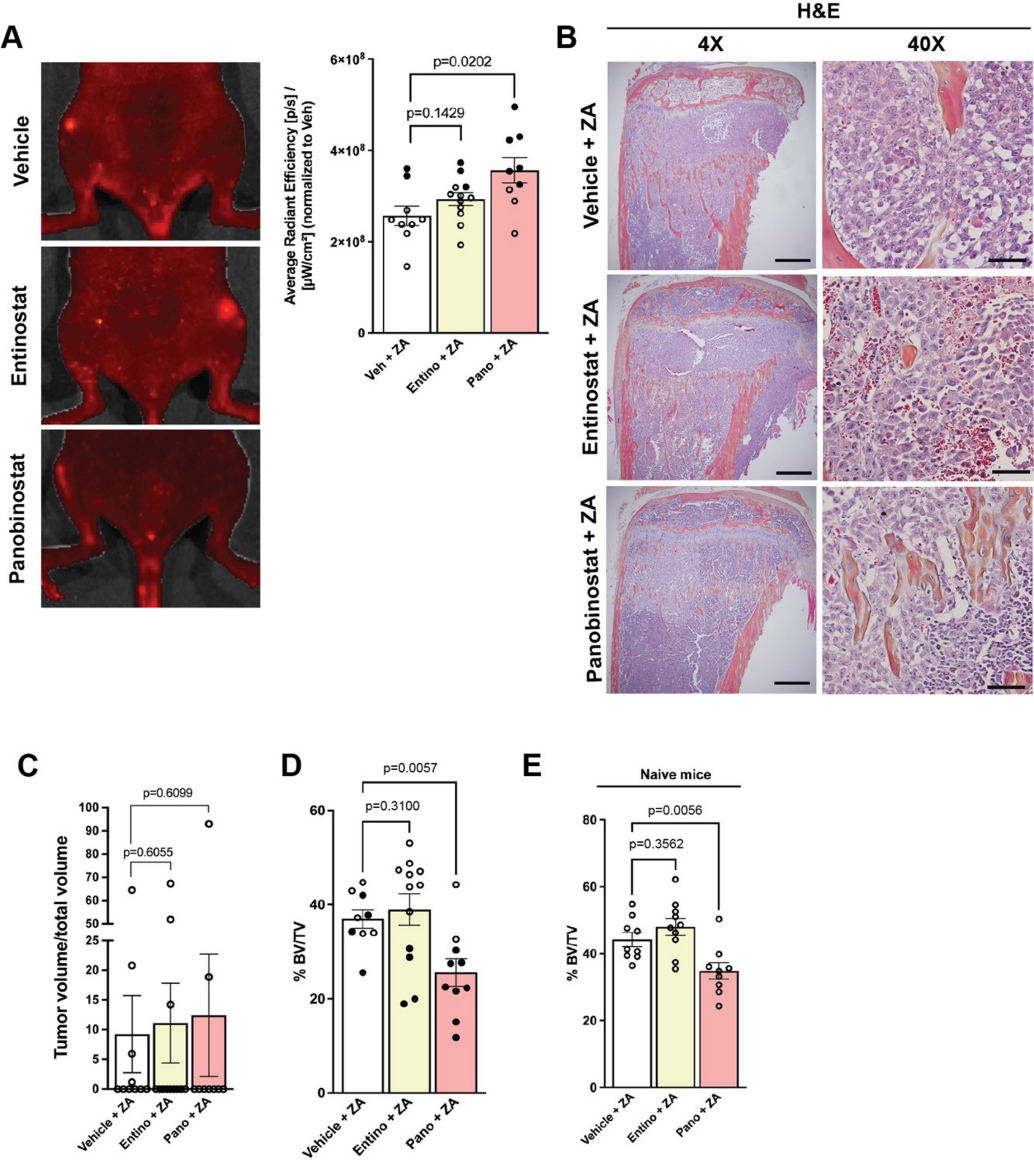
Combination treatment of entinostat with bisphosphonate prevents bone metastatic progression. (*A*) Representative *in vivo* bioluminescence images and quantitation at endpoint of luciferase‐expressing MDA‐MB‐231b‐inoculated mice treated with vehicle + zoledronic acid (ZA), entinostat + ZA, or panobinostat + ZA (*n* = 10–12 mice/group). (*B*) Representative hematoxylin and eosin (H&E) images of tibiae from mice described in (*A*). 4×, scale bar = 500 μM; 40×, scale bar = 100 μM. (*C*) Histologic analysis of tumor cells in the bone marrow of mice described in (*A*, *B*). (*D*) Micro‐CT analysis of bone volume/total volume (%BV/TV) of mice described in (*A*). (*E*) micro‐CT analysis of bone volume/total volume (%BV/TV) of tumor‐naïve mice treated with vehicle + ZA, entinostat + ZA, or panobinostat + ZA (*n* = 9–10 mice/group). Mice presented in Fig. 1 represent tumor‐naïve mice and in Fig. 2 represent tumor‐inoculated mice without ZA that can be directly compared with the mice presented herein as they were all included in the same experiment.

Because we observed a significant increase in pro‐osteolytic genes in breast cancer cells treated with entinostat, and bisphosphonates are FDA‐approved for metastatic breast cancer and improve survival for postmenopausal patients with breast cancer,^(^ ^27^, ^28^ ^)^ we investigated whether combination of entinostat with bisphosphonate would prevent the progression of bone metastases and prevent bone loss caused by panobinostat in the bone metastatic setting. Mice were inoculated with MDA‐MB‐231b cells by intracardiac injection, given one dose of the bisphosphonate zoledronic acid the next day, and treated with entinostat or panobinostat for 4 weeks. *In vivo* bioluminescence imaging for luciferase‐positive MDA‐MB‐231b tumor cells revealed significantly higher tumor burden in mice treated with panobinostat, but not entinostat, compared with vehicle‐treated mice (1.4‐fold, *p* = 0.0202; Fig. 5*A*). However, analysis of tumor burden by histomorphometry found no significant difference in tumor burden in mice treated with a combination of entinostat + zoledronic acid or panobinostat + zoledronic acid compared with vehicle + zoledronic acid‐treated mice (Fig. 5*B*, *C*). These data indicate that zoledronic acid generally prevents the expansion of tumor burden in bone caused by entinostat and potentially panobinostat treatment (Fig. 2). There was no significant difference in bone volume between vehicle + zoledronic acid and entinostat + zoledronic acid‐treated mice (Fig. 5*D*), similar to that observed with entinostat treatment alone in the tumor‐naïve model (Fig. 1*B*, *F*) and the bone metastatic model (Fig. 2*E*). However, bone volume remained significantly lower in tumor‐bearing mice treated with panobinostat + zoledronic acid compared with vehicle + zoledronic acid‐treated mice (1.44‐fold, *p* = 0.0057; Fig. 5*D*). Although the overall bone volume in tumor‐bearing mice was significantly higher with panobinostat + zoledronic acid (Fig. 5*D*) compared with panobinostat treatment alone (25% versus 2.5%, *p* < 0.0001, Fig. 2*E*), these data suggest that panobinostat detrimentally impacts trabecular bone volume in the bone metastatic setting, and this persists when bone resorption is inhibited by a bisphosphonate. We also confirmed in tumor‐naïve mice that there is no difference in bone volume in mice treated with entinostat + zoledronic acid compared with vehicle + zoledronic acid treatment (Fig. 5*E*). However, there is significantly lower bone volume in tumor‐naïve mice treated with panobinostat + zoledronic acid alone compared with vehicle + zoledronic acid treatment (1.27‐fold, *p* = 0.0056; Fig. 5*E*). Thus, in all settings tested, treatment with panobinostat resulted in significantly reduced bone volume compared with vehicle treatment, even with the addition of zoledronic acid.

## Discussion

**Fig. 6:**
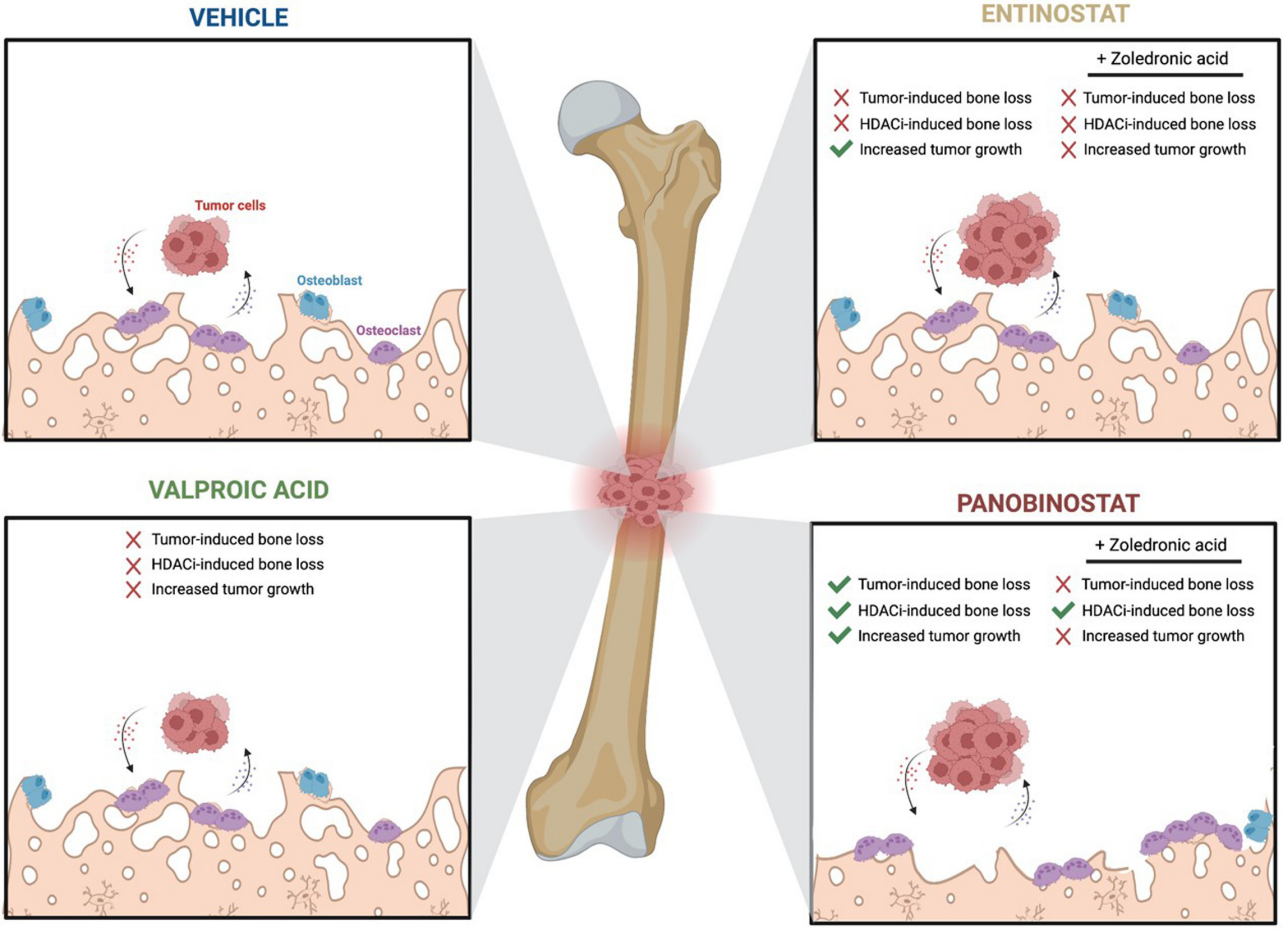
Impact of HDACi alone and in combination with zoledronic acid on the bone and metastatic tumors. Working model depicting changes that occur to the bone microarchitecture and tumor growth in response to valproic acid, entinostat, and panobinostat treatment. Zoledronic acid corrects HDACi‐enhanced tumor growth but not panobinostat‐induced bone loss. Figure made with BioRender.

While breast cancer frequently metastasizes to the bone, therapeutic avenues to reduce metastatic incidence and tumor burden remain limited. The effects of HDACi on bone‐disseminated tumor cells and the mechanisms involved warrant investigation given that these therapies are currently being tested in clinical trials for metastatic breast cancer. Previous evidence indicates that HDACi, specifically valproic acid and vorinostat, cause bone loss in numerous mouse strains.^(^ ^13^, ^14^ ^)^ Our data indicate that panobinostat, but not entinostat or valproic acid, significantly impacts bone volume, trabecular number, and trabecular spacing. This bone loss was only partially mitigated with the addition of zoledronic acid to panobinostat treatment. In the context of bone metastasis, treatment with panobinostat and entinostat, but not valproic acid, resulted in higher tumor burden compared with vehicle‐treated mice, which was corrected by zoledronic acid. However, the combination of zoledronic acid and panobinostat failed to correct the HDACI‐induced bone loss (Fig. 6). These findings suggest that HDACi‐mediated bone destruction may not be coupled with growth of bone metastases.

It is important to note that numerous studies, predominantly investigating primary tumor growth, have shown the beneficial effects of HDACi in reducing breast tumor growth. Entinostat specifically has been shown to inhibit tumor growth and prolong survival in numerous preclinical models of breast cancer irrespective of molecular subtype.^(^ ^29^, ^30^ ^)^ Many mechanisms of HDACi‐mediated antitumor effects have been demonstrated, including repression of Myc target genes, induction of apoptotic and autophagic cell death, cell cycle arrest, and accumulation of DNA damage.^(^ ^29^, ^31^, ^32^, ^33^ ^)^ In contrast to these primary tumor studies, our findings demonstrate that metastatic growth in the bone is significantly elevated after some HDACi treatments, including entinostat. Recent clinical trials using HDACi in combination with the estrogen modulator exemestane showed either only a small improvement in progression‐free survival (PFS)^(^ ^34^ ^)^ or no PFS advantage in patients with metastatic ER^+^ breast cancer.^(^ ^35^ ^)^ Similarly, combination therapy of an HDACi with the DNA methyltransferase inhibitor 5‐azacitidine showed no significant improvement in metastatic tumor response.^(^ ^36^ ^)^ Given these findings and the lack of bone destruction after entinostat treatment, we hypothesize that entinostat may upregulate factors within bone‐disseminated tumor cells that promote their progression in bone, without inducing significant bone destruction. This enhanced HDACi‐mediated bone resorption may perpetuate the tumor‐induced bone disease and tumor progression through the release of pro‐tumorigenic factors from the bone matrix but in localized pockets. In support of this hypothesis, our findings indicate that multiple HDACi induce expression of pro‐osteolytic factors in tumor cells. Notably, in addition to its effects on osteolysis, upregulation of PTHrP directly promotes tumor growth by promoting entry into the cell cycle.^(^ ^24^ ^)^ Together, these findings provide a potential mechanism to explain why HDACi promote the growth of bone metastases while reducing primary breast tumor growth.

The mechanism by which zoledronic acid prevents entinostat and panobinostat‐induced tumor progression in bone remains unknown but may be partially attributable to known antitumor effects of zoledronic acid. Although it continues to be debated in the field, zoledronic acid directly reduces breast cancer cell proliferation, inhibits cell migration, and induces apoptosis.^(^ ^37^, ^38^, ^39^ ^)^ A recent study showed that zoledronic acid treatment reduced DNA synthesis, induced cell cycle arrest, and programmed cell death in numerous breast cancer cell lines.^(^ ^38^ ^)^ Zoledronic acid also modulates the interaction between MDA‐MB‐231 cells and regulatory T cells, resulting in reduced regulatory T‐cell proliferation, migration, and immunosuppressive functions.^(^ ^40^ ^)^ Additionally, zoledronic acid can be engulfed by tumor‐associated macrophages within primary breast tumors, which may also contribute to the antitumor activity of zoledronic acid.^(^ ^41^ ^)^ Notably, many studies investigating zoledronic acid in breast cancer have been performed using in vitro models; thus, the direct effects of zoledronic acid on breast cancer cells in mouse models of metastasis remain somewhat unclear.

Our results indicate that patients would likely not benefit from treatment with entinostat or panobinostat alone for bone metastases, and treatment with either drug alone may in fact worsen tumor progression in bone, potentially through increased expression of pro‐osteolytic factors and resulting osteolysis. Importantly, the addition of zoledronic acid mitigates the pro‐tumorigenic effect of both entinostat and to a lesser extent panobinostat on tumor progression in bone, suggesting patients with bone metastatic disease or at elevated risk of developing bone metastases might benefit from the addition of zoledronic acid. However, although zoledronic acid corrects the impact on tumor progression, panobinostat‐induced bone loss persists, suggesting that entinostat may be more “bone‐sparing.” Beyond bone metastasis, our findings are highly relevant to those patients treated with HDACi for a primary tumor who likely harbor disseminated tumor cells in their bone. Tumor cells disseminate to distant sites early in tumor progression^(^ ^42^, ^43^ ^)^ and lie dormant before forming clinically overt metastases. Although entinostat or panobinostat treatment may reduce primary tumor growth, they may also inadvertently fuel tumor growth in the bone. Our findings demonstrate a critical need to better understand HDACi effects on tumor outgrowth in the bone in the context of both primary and metastatic breast cancer.

## Disclosures

The authors declare no competing financial interests.

## AUTHOR CONTRIBUTIONS

**Miranda E Clements**: Conceptualization; data curation; formal analysis; investigation; methodology; visualization; writing – original draft; writing – review and editing. **Lauren Holtslander**: Data curation; formal analysis; investigation; methodology. **Joshua R Johnson**: Data curation; investigation; methodology. **Rachelle W Johnson**: Conceptualization; data curation; formal analysis; funding acquisition; investigation; methodology; project administration; resources; supervision; visualization; writing – original draft; writing – review and editing.

### PEER REVIEW

The peer review history for this article is available at https://publons.com/publon/10.1002/jbm4.10694.

## Data Availability

Data are displayed as individual points on each graph. Source data for all experiments is available upon reasonable request.
